# Distinct iPS Cells Show Different Cardiac Differentiation Efficiency

**DOI:** 10.1155/2013/659739

**Published:** 2013-10-27

**Authors:** Yohei Ohno, Shinsuke Yuasa, Toru Egashira, Tomohisa Seki, Hisayuki Hashimoto, Shugo Tohyama, Yuki Saito, Akira Kunitomi, Kenichiro Shimoji, Takeshi Onizuka, Toshimi Kageyama, Kojiro Yae, Tomofumi Tanaka, Ruri Kaneda, Fumiyuki Hattori, Mitsushige Murata, Kensuke Kimura, Keiichi Fukuda

**Affiliations:** ^1^Department of Cardiology, Keio University School of Medicine, Tokyo 160-8582, Japan; ^2^Biomedical Research Laboratories, Asubio Pharma Co., Ltd., Kobe 650-0047, Japan

## Abstract

Patient-specific induced pluripotent stem (iPS) cells can be generated by introducing transcription factors that are highly expressed in embryonic stem (ES) cells into somatic cells. This opens up new possibilities for cell transplantation-based regenerative medicine by overcoming the ethical issues and immunological problems associated with ES cells. Despite the development of various methods for the generation of iPS cells that have resulted in increased efficiency, safety, and general versatility, it remains unknown which types of iPS cells are suitable for clinical use. Therefore, the aims of the present study were to assess (1) the differentiation potential, time course, and efficiency of different types of iPS cell lines to differentiate into cardiomyocytes in vitro and (2) the properties of the iPS cell-derived cardiomyocytes. We found that high-quality iPS cells exhibited better cardiomyocyte differentiation in terms of the time course and efficiency of differentiation than low-quality iPS cells, which hardly ever differentiated into cardiomyocytes. Because of the different properties of the various iPS cell lines such as cardiac differentiation efficiency and potential safety hazards, newly established iPS cell lines must be characterized prior to their use in cardiac regenerative medicine.

## 1. Introduction

Embryonic stem (ES) cells are attractive candidates for use in cardiovascular stem cell-based therapy because mouse and human ES cells have been shown to have the capacity for unlimited proliferation and promising multipotency [1, 2]. Evidence also indicates that ES cells are one of the best candidates for use in cell-replacement therapy for cardiac diseases because of their ability to differentiate and proliferate, supplying a substantial number of mature human cardiac myocytes for transplantation into large, diseased human hearts [3–5]. Previous studies in animal models have shown that transplantation of ES cell-derived cardiac myocytes improves cardiac function and survival [6, 7]. However, the establishment and use of human ES cells remain contentious on ethical and legal grounds because of the origin of ES cells as well as concerns regarding immunological rejection or the need for immunosuppressant drugs after cell transplantation [8].

Mouse and human induced pluripotent stem (iPS) cells are artificially established pluripotent stem cells that resemble ES cells [9–16]. The iPS cells are similar to ES cells in terms of their morphology, proliferative ability, surface antigens, gene expression, epigenetic status of pluripotent stem cell-specific genes, and telomerase activity. Moreover, mouse iPS cells have exhibited germline contribution and tetraploid complementation, which are among the most desirable stem cell characteristics [10, 13, 17]. Human iPS cells were initially generated from adult skin fibroblasts by the gene transfer of four transcription factors (*Oct3/4*, *Sox2*, *Klf4*, and *c-Myc*); however, recent evidence indicates that several other types of somatic cells can be used to generate iPS cells with or without residual transgene [18]. Because iPS cells can be easily generated from human somatic cells and have almost the same potential as ES cells, it should be possible to develop iPS cell-derived cardiac myocyte transplantation as a treatment for the failing heart, without the ethical issues or immune rejection associated with the use of ES cells.

Therefore, iPS cells are regarded as powerful candidates for stem cell-based cell-replacement therapy, with the expectation that their use may address many remaining challenges in cardiac regenerative medicine for severe heart diseases which currently have a very poor prognosis. Although the morphology, growth characteristics, and pluripotency of iPS cells are believed to be similar to those of ES cells, it is not clear whether every iPS cell line has similar properties and is suitable for use in cardiac regenerative medicine. In the present study, we used the well-characterized iPS cell lines Fbx15 and Nanog. The Fbx15-iPS cells were the first reported iPS cell line and they exhibit similar morphology, proliferation, and teratoma formation to ES cells, although they do not exhibit germline competency [9]. The Nanog-iPS cells were subsequently generated to overcome these problems, and this cell line exhibits successful transmission through the germline to the next generation [13]. Therefore, in the present study we used Fbx15-iPS cells as low-quality iPS cells and Nanog-iPS cells as high-quality iPS cells. Current concerns for cell transplantation therapy using iPS cells are focused on tumor formation following the transplantation of iPS cell-derived cells. Specifically, the presence of residual transgenes in the differentiated cells and contamination of iPS cells within the differentiated cells are of concern. However, there have been few attempts to clarify whether differentiated cardiomyocytes from the various types of iPS cells exhibit normal function and/or identical function to the ES cell-derived cardiomyocytes. To realize the potential of iPS cells for use in regenerative medicine for the treatment of various cardiac diseases, we need to determine the best type of iPS cells to use. Thus, the aims of the present study were to (1) elucidate the differences in cardiomyocyte differentiation potential among several mouse iPS cell lines and (2) perform a molecular characterization of the cardiomyocytes derived from the different iPS cell lines.

## 2. Materials and Methods

### 2.1. Mouse ES and iPS Cells

Mouse ES cells (EB3) were obtained from the Laboratory of Pluripotent Cell Studies, RIKEN Center for Developmental Biology. Mouse iPS cells (iPS-MEF-Ng-20D17, 38C2, 38D2, iPS-TTF-Fbx-WT-1, and 4-3) were obtained from the Center for iPS Cell Research and Application, Kyoto University, and were used in accordance with the Guidelines for Derivation and Utilization of Human Embryonic Stem Cells of the Ministry of Education, Culture, Sports, Science, and Technology, Japan. Nanog-iPS, Fbx15-iPS, and ES cells were used to compare the characteristics between different iPS cell lines and ES cells. To generate Fbx15-iPS cells, transgenes were introduced into mouse tail tip fibroblasts by a retrovirus carrying *Oct3/4 *(also called *Pou5f1*), *Sox2*, *Klf4*, and *c-Myc. *Subsequently, Fbx15-iPS cell colonies were selected by innate* Fbx15* (also called *Fbxo15*) expression, in which selection marker genes were knocked-in under the innate Fbx15 promoter [9]. To generate Nanog-iPS cells, transgenes were introduced into embryonic fibroblasts of bacterial artificial chromosome (BAC) transgenic mice by a retrovirus carrying *Oct3/4*, *Sox2*, *Klf4*, and *c-Myc.* Subsequently, Nanog-iPS cells were selected by Nanog promoter activation, in which the selection marker gene was inserted under the Nanog promoter, which was incorporated in the BAC transgene [13]. We confirmed that the iPS cells exhibited typical ES cell-like features and all cells were morphologically similar, and staining for alkaline phosphatase (ALP), a marker of undifferentiated cells, revealed strong ALP expression in ES, Nanog-iPS, and Fbx15-iPS cells.

### 2.2. Maintenance of Mouse ES and iPS Cells

Mouse ES and iPS cells were maintained on gelatin-coated dishes in Glasgow minimum essential medium (Sigma-Aldrich, St. Louis, MO) supplemented with 10% fetal bovine serum (FBS; Equitech-Bio Inc, Kerrville, TX), 0.1 mM minimum essential medium (MEM) nonessential amino acids solution (Sigma-Aldrich, St. Louis, MO), 2 mM l-glutamine (Sigma-Aldrich, St. Louis, MO), 0.1 mM *β*-mercaptoethanol (Sigma-Aldrich, St. Louis, MO), and 2000 U/mL murine leukemia inhibitory factor (LIF; Millipore, Billerica, MA). Fbx15-iPS cells were maintained on mouse embryonic fibroblasts in Dulbecco's modified Eagle's medium (Life Technologies, Grand Island, NY) supplemented with 10% FBS (Equitech-Bio Inc, Kerrville, TX), 0.1 mM MEM non-essential amino acids solution (Sigma-Aldrich, St. Louis, MO), 2 mM l-glutamine (Sigma-Aldrich, St. Louis, MO), 0.1 mM *β*-mercaptoethanol (Sigma-Aldrich, St. Louis, MO), and 2000 U/mL murine LIF (Millipore, Billerica, MA).

### 2.3. Differentiation of Mouse ES and iPS Cell-Derived Cardiomyocytes

Mouse ES and iPS cells were harvested with 0.25% trypsin-EDTA and dissociated. Four hundred cells were used to form one EB in one hanging drop with *α*-modified Eagle's medium (*α*MEM; Sigma-Aldrich, St. Louis, MO) supplemented with 10% FBS (Equitech-Bio Inc, Kerrville, TX). On day 2, the EBs were transferred into floating culture plates with new medium. Then, 4-5 days after differentiation, floating EBs were transferred into attachment cultures. Typically, beating cells appeared on day 7.

### 2.4. Immunofluorescent Staining

Cells were fixed with 4% paraformaldehyde in phosphate-buffered saline (pH 7.0) for 20 min. Subsequently, cells were permeabilized with 0.2% Triton X-100 (Sigma-Aldrich, St. Louis, MO) at room temperature for 10 min and then incubated with the primary antibody at 4°C overnight. Cells were washed with Tris-buffered saline containing 0.1% Tween-20 four times prior to incubation with the secondary antibodies at room temperature for 30 min. The primary antibodies used in the present study were mouse monoclonal anti-*α*-*actinin* (1 : 200 dilution; Sigma-Aldrich, St. Louis, MO) and anti-myosin heavy chain (MF20; 1 : 100; Developmental Studies Hybridoma Bank), goat polyclonal anti-*Nkx2.5* (1 : 50; Santa Cruz Biotechnology) and anti-*GATA4* (1 : 50; Santa Cruz Biotechnology), and rabbit polyclonal anti-ANP (1 : 200; Phoenix Pharmaceuticals). Cells were incubated with the fluorescent dye-conjugated secondary antibodies for 30 min at room temperature. After nuclear staining with 4′,6′-diamidino-2-phenylindole (Life Technologies, Grand Island, NY), fluorescence signals were observed under a fluorescence microscope (IX71; Olympus, Japan).

### 2.5. Total RNA Extraction, cDNA Synthesis, and Real-Time PCR

Total RNA was prepared from tissues and embryoid bodies using ISOGEN (Nippon Gene, Japan) according to the manufacturer's instructions. Contaminating genomic DNA was degraded by RNase-Free DNase I (Life Technologies, Grand Island, NY) at 37°C for 30 min. After treatment, DNase was inactivated by phenol-chloroform extraction and ethanol precipitation. We reverse transcribed total RNAs into cDNA using the oligo-(dT)12–18 primer (Superscript II RT kit; Life Technologies, Grand Island, NY). The RT-PCR was performed as described previously [19]. The target gene names and identification numbers of the primer and probe mixtures (Applied Biosystems) are given in the Supplementary Table 1, available online at http://dx.doi.org/10.1155/2013/659739. 

### 2.6. Electrophysiology

Electrophysiological studies were performed using a microscope equipped with a recording chamber and a noise-free heating plate (Microwarm Plate; Kitazato Supply). HEPES (10 mmol/L) was added to the culture medium to maintain the pH of the perfusate at 7.5-7.6. Standard glass microelectrodes that had a DC resistance of 25–35 M*Ω* when filled with pipette solution (2 mol/L KCl) were used. The electrodes were positioned using a motor-driven micromanipulator (EMM-3SV; Narishige, Japan) under optical control. Spontaneously beating cells were selected as targets, and the action potentials of the targeted cells were recorded. The recording pipette was connected to a patch-clamp amplifier (Axopatch 200B; Axon Instruments); signals were passed through a low-pass filter with a cutoff frequency of 2 kHz and digitized with an A/D converter with a sampling frequency of 10 kHz (Digidata 1440A; Axon Instruments). Signals were monitored, recorded as electronic files, and analyzed offline with pCLAMP 10 software (Axon Instruments).

### 2.7. Field Potential Recordings Using the On-Chip MEA System

The MEA chips were obtained from Multi Channel Systems (Germany) and were coated with fibronectin (F1141 Sigma-Aldrich, St. Louis, MO). Beating EBs were plated on the electrodes and incubated at 37°C in a humidified atmosphere of 95% air and 5% CO_2_. MEA measurements were performed at 37°C. The signals were initially processed, and the data obtained were subsequently analyzed with MC_Rack (Multi Channel Systems). Data for analysis were extracted from the 2 min mark to the 5 min of recording. Isoproterenol and verapamil (Sigma-Aldrich, St. Louis, MO) were dissolved in distilled water as 0.1, 1, and 10 mM and 10 *μ*M stock solutions. These stock solutions were diluted to the required concentration in culture medium, and applied to the test chip. The protocol for the drug assays was as follows: the medium on the test chip was exchanged for fresh medium, and field potentials were recorded for 5 min. Subsequently, the drug was applied to the test chip and, after 5–10 min incubation, field potentials were measured again.

### 2.8. Statistical Analysis

Results are reported as the mean ± SEM. Student's *t*-test was used to evaluate the significance of any differences. *P* < 0.05 was considered significant.

## 3. Results

### 3.1. Fbx15-iPS Cells Show Significant Residual Transgene Expression

**Figure 1:**
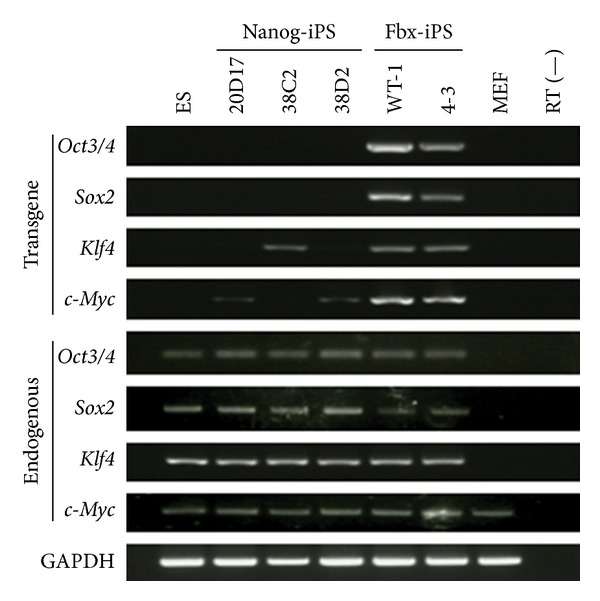
Endogenous pluripotent gene and transgene expression profiles of undifferentiated pluripotent cells. Expression levels of the four transcription factors. Semiquantitative RT-PCR analyses were performed for endogenous pluripotent stem cell transcription factors and transgenes.

To assess the molecular features of ES, Nanog-iPS, and Fbx15-iPS cells, the expression of four endogenous and exogenous (transgene) transcription factors was examined by semiquantitative RT-PCR. The results showed similar expression of the four endogenous transcription factors in the ES, Nanog-iPS, and Fbx-iPS cells. Two independent Fbx-iPS cell lines, namely, WT-1 and 4-3, clearly showed residual transgene expression. The expression of the four transgenes was significantly lower in three independent Nanog-iPS cell lines (i.e., 20D17, 38C2, and 38D2) than in Fbx15-iPS cells (Figure 1). Two of these Nanog-iPS cell lines, namely, 20D17 and 38D2, showed c-Myc transgene expression, whereas the Nanog-iPS cell line 38C2 showed Klf4 transgene expression. Different expression of residual transgenes was observed in three Nanog-iPS cell lines, and there was no unified pattern of transgene expression among the iPS cells. These findings suggest that retroviral transgene expression is largely silenced in Nanog-iPS cells compared with Fbx 15-iPS cells, and that the degree of transgene silencing varies among the Nanog-iPS cell lines. It is generally considered that partial reprogrammed iPS cells express the viral transgenes and endogenous pluripotency genes, and full-reprogrammed iPS cells silence the viral transgenes as the endogenous genes maintain the pluripotent state [20]. Thus, retroviral vector silencing could serve as a quality indicator for the iPS cells and high-quality iPS cells silence transgene and low-quality iPS cells express transgene.

### 3.2. Nanog-iPS Cells Exhibit Greater Beating Efficiency Than Fbx15-iPS Cells

**Figure 2:**
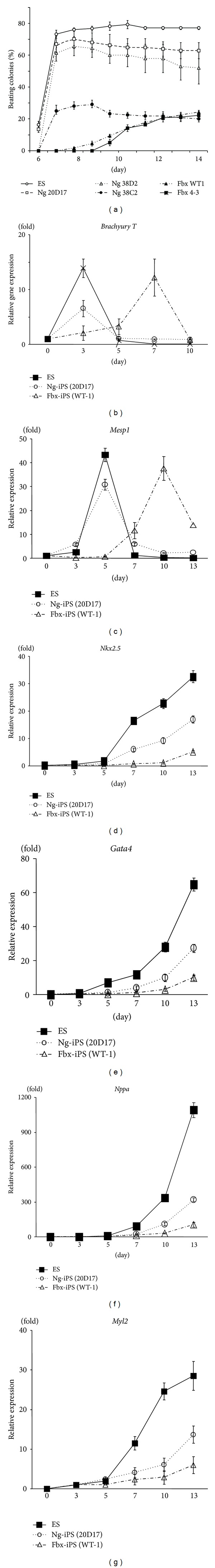
Cardiomyocyte differentiation efficiency of pluripotent stem cells and temporal gene expression patterns during cardiomyocyte differentiation. (a) Percentage of beating colonies on days 6–15 in mouse embryonic stem (ES) cells, Nanog induced pluripotent stem (iPS) cells, and Fbx15-iPS cells. Data are the mean ± SEM (*n* = 5 in all groups). ((b)–(g)) Quantitative RT-PCR analyses showing temporal gene expression patterns of the mesodermal marker Brachyury *T* (b), the early cardiac mesodermal marker *Mesp1* (c), the cardiac-specific transcription factors *Nkx2.5* (d) and *Gata4* (e), and the cardiac-specific proteins *Nppa* (f) and *Myl2* (g) in EB3 ES cells (closed squares), 20D17 Nanog-iPS cells (open circles), and WT-1 Fbx15-iPS cells (open triangles). Data are the mean ± SEM (*n* = 5 in all groups).

To examine differences in the efficiency of cardiac differentiation, the conventional hanging drop method was used to induce spontaneously beating embryoid bodies (EBs), which contain a rich population of cardiomyocytes. Beating efficiency of EBs is well correlated with cardiomyocyte differentiation from ES and iPS cells and can be a good marker for cardiomyocyte differentiation efficiency [5, 21]. Around day 6-7 after EB formation, beating EBs started to be seen in the ES cells and in all Nanog-iPS cell lines. However, it took longer for the Fbx15-iPS cell lines to differentiate into beating EBs (Figure 2(a)). Furthermore, the incidence of beating EBs was significantly lower in the Fbx15-iPS cell lines. Interestingly, the beating efficiency differed between the three Nanog-iPS cell lines evaluated. Specifically, the incidence of beating EBs was significantly higher in the 20D17 and 38D2 Nanog-iPS cell lines than in both the 38C2 Nanog-iPS cell line and the Fbx15-iPS cell lines, although the incidence of beating EBs remained significantly lower than that in ES cells. There were no significant differences between the 38C2 Nanog-iPS cell line and the Fbx15-iPS cell lines after day 12, although there were differences in the time until spontaneous beating was observed in the 38C2 Nanog-iPS cell line and Fbx15-iPS cell lines. These results indicate that there are differences in the efficiency of cardiac differentiation as well as in the time required for cardiac differentiation, between individual iPS cell lines.

### 3.3. Expression of Mesodermal and Cardiac Marker Genes Is Significantly Decreased and Delayed in Fbx15-iPS Cells

The Fbx15-iPS cells expressed transgenes and exhibited a lower efficiency of cardiac differentiation than ES and Nanog-iPS cells. To determine which step is impeded during the cardiac differentiation of Fbx15-iPS cells from pluripotent stem cells, we evaluated the pattern of temporal gene expression during cardiomyocyte differentiation. The temporal expression of *Brachyury T*, a mesodermal marker, was similar in ES and Nanog-iPS cells. In contrast, *Brachyury T* expression was significantly delayed in Fbx15-iPS cells, which suggests that these cells tend to resist mesodermal differentiation (Figure 2(b)). The expression of *Mesp1*, one of the earliest cardiac fate markers, was transiently upregulated on days 3 and 5 in ES and Nanog-iPS cells; however, *Mesp1* expression was significantly delayed in Fbx15-iPS cells (Figure 2(c)). In terms of cardiac-specific genes, we evaluated the expression of the cardiac transcription factors *Nkx2.5* and *Gata4* as well as that of the cardiac-specific genes *natriuretic peptide type A (Nppa)* and *myosin light chain-2v (Myl2)*. For all cardiac-specific genes, there was tendency for highest expression to be detected in ES cells, with the lowest (and delayed) expression seen in Fbx15-iPS cells (Figures 2(d)–2(g)). These data suggest that Fbx15-iPS cells exhibit resistance to mesodermal and cardiac differentiation.

### 3.4. Purified Fbx15-iPS Cell-Derived Cardiomyocytes Retain Residual Transgene Expression

**Figure 3:**
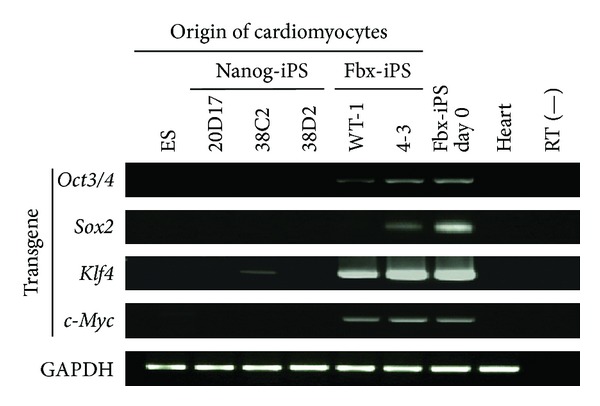
Transgene expression profiles of purified cardiomyocytes derived from Nanog and Fbx15 induced pluripotent stem (iPS) cells. Total RNA was isolated from purified cardiomyocytes derived from mouse embryonic stem (ES) cells, three Nanog-iPS cell clones (20D17, 38C2, and 38D2), two Fbx15-iPS cell clones (WT-1 and 4-3), undifferentiated Fbx15-iPS cells, and the mouse heart. RT-PCR analyses were performed to determine the transgene expression of the four transcription factors (*Oct3/4*, *Sox2*, *Klf4*, and *c-Myc*), with GAPDH used as an internal control.

As described above, Fbx15-iPS cells continue to express residual transgenes and have attenuated cardiac differentiation efficiency. Despite this, beating cardiomyocytes were obtained from Fbx15-iPS cells. This may be due to heterogeneous Fbx15-iPS cell populations: despite the presence of transgene-positive iPS cells that are resistant to differentiation, transgene-negative iPS cells are able to differentiate into cardiomyocytes. To determine whether Fbx15-iPS cell-derived cardiomyocytes express transgenes after differentiation, we purified cardiomyocytes using the tetramethylrhodamine methyl ester (TMRM) fluorescent dye method [22]. Surprisingly, even after purification of Fbx15-iPS cell-derived cardiomyocytes, expression of residual transgenes remained relatively high (Figure 3). Moreover, among the three Nanog-iPS cell lines, the 38C2 cells were found to have low levels of expression of the Klf4 residual transgene. These data suggest that transgene-positive iPS cells can differentiate into cardiomyocytes, and that transgenes are still expressed after cardiac differentiation. Together, the observations that the Fbx15-iPS cell lines have a low cardiac differentiation efficiency and that the 38C2 Nanog-iPS cell line has a lower beating efficiency than the other Nanog-iPS cell lines suggest that residual expression of the *Klf4* transgene may have a negative effect on cardiomyocyte differentiation. Furthermore, the relatively high expression of partially silenced retroviral transgenes may explain the differences in the efficiency of cardiomyocyte differentiation between the ES, Nanog-iPS, and Fbx15-iPS cells.

### 3.5. Cardiac-Specific Marker Gene Expression in iPS Cell-Derived Cardiomyocytes

**Figure 4:**
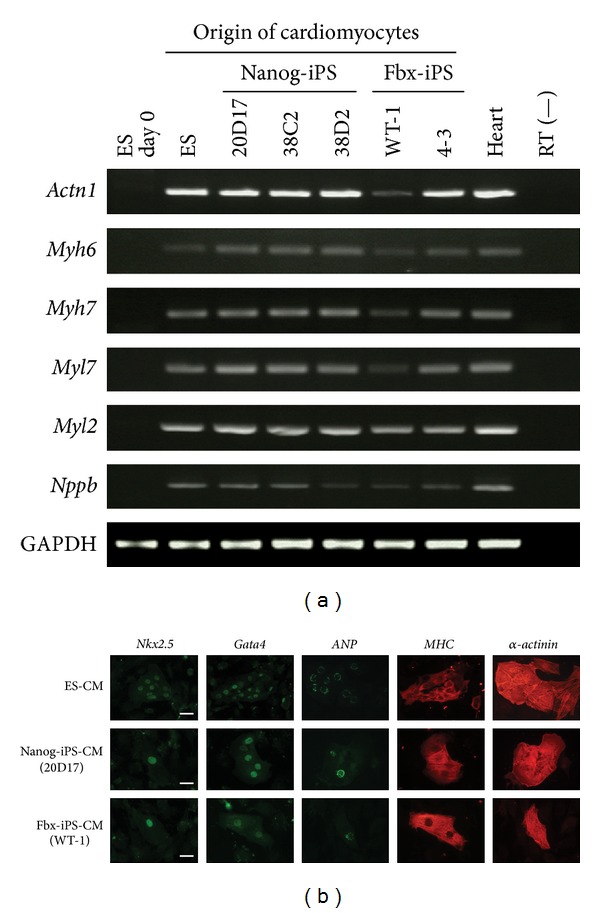
Cardiomyocyte (CM) specific gene expression profiles, as determined by RT-PCR and immunostaining, of CM derived from mouse embryonic stem (ES) cells, Nanog induced pluripotent stem (iPS) cells, and Fbx15-iPS cells. (a) CM-associated structural protein gene expression profiles. The expression of *Actn1*, *Myh6*, *Myh7*, *Myl7*, *Myl2*, and* Nppb* was analyzed by semiquantitative RT-PCR analysis. GAPDH was used as an internal control. (b) Immunofluorescent staining of typical CM-specific proteins on day 15 of differentiation in CM derived from ES, Nanog-iPS, and Fbx15-iPS cells. Cells were stained with *Nkx2.5* (green), *GATA4* (green), atrial natriuretic peptide (*ANP*; green), myosin heavy chain (*MHC*; red), and *α*-*actinin* (red). Scale bar = 50 *μ*m.

Because iPS cell-derived cardiomyocytes continue to express transgenes at various levels, semiquantitative RT-PCR and immunostaining were used to evaluate the expression of cardiac-specific genes in ES, Nanog-iPS, and Fbx15-iPS cell-derived cardiomyocytes. As shown in Figure 4(a), RT-PCR analysis revealed the appropriate expression of various cardiac marker genes, including *α myosin heavy chain* (*Myh6*), *β myosin heavy chain* (*Myh7*), *myosin light chain-2a* (*Myl7*), *Myl2*, *α-sarcomeric actinin* (*Actn1*), and *natriuretic peptide type B* (*Nppb*). Immunofluorescence analyses revealed that the ES and iPS cell-derived cardiomyocytes showed typical sarcomere formation based on positive staining with antibodies directed against *α*-actinin and myosin heavy chain (MHC), proper nuclear expression of the cardiac-specific transcription factors *Nkx2.5* and *GATA4*, and the presence of atrial natriuretic peptide (ANP) in the secretory granules surrounding the nuclei (Figure 4(b)).

### 3.6. Electrophysiological Demonstration of Normal Functional Properties of iPS Cell-Derived Cardiomyocytes

**Figure 5:**
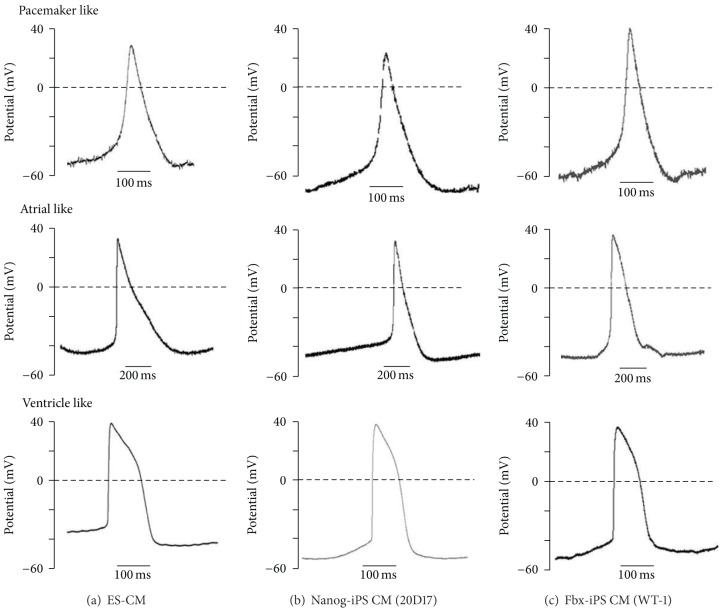
Electrophysiological studies of cardiomyocytes (CM) derived from mouse embryonic stem (ES) cells, Nanog induced pluripotent stem (iPS) cells, and Fbx15-iPS cells. Representative action potentials of (a) ES cell-derived CM, (b) Nanog-iPS cell-derived CM, and (c) Fbx15-iPS cell-derived CM showing sinus node-like (top), fetal atrial-type (middle), and fetal ventricular-type (bottom) action potentials.

Because iPS cell-derived cardiomyocytes continue to express transgenes at various levels, we recorded the action potentials of iPS cell-derived cardiomyocytes using glass microelectrodes to characterize the electrophysiological properties of iPS cell-derived cardiomyocytes. ES cell-derived cardiomyocytes were used as a control. Day 20 beating colonies were selected and dispersed into small clusters and single cells. Pacemaker-like, atrial-like, and fetal ventricular-type action potentials were recorded in ES and iPS cell-derived cardiomyocytes (Figures 5(a)–5(c)) [23, 24]. Both Nanog-iPS and Fbx15-iPS cell-derived cardiomyocytes exhibited appropriate pacemaker-like, atrial-like, and fetal ventricular-type action potentials.

### 3.7. Normal Chronotropic Responses of iPS Cell-Derived Cardiomyocytes

**Figure 6:**
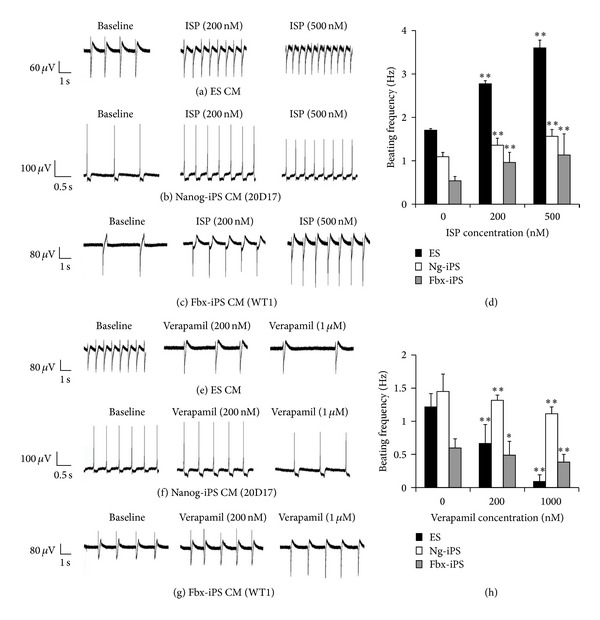
Pharmacological studies of cardiomyocytes (CM) derived from mouse embryonic stem (ES) cells, Nanog induced pluripotent stem (iPS) cells, and Fbx15-iPS cells. ((a)–(c)) Multielectrode array (MEA) recordings in CM derived from ES (a), Nanog-iPS (b), and Fbx15-iPS cells (c) showing chronotropic responses to increasing doses of isoproterenol. (d) Summary of the positive chronotropic changes induced by isoproterenol. Data are the mean ± SEM. *P < 0.05 and **P < 0.01 compared with 0 mM isoproterenol. ((e)–(g)) MEA recordings of negative chronotropic responses to increasing doses of verapamil in CM derived from ES (d), Nanog-iPS (e), and Fbx15-iPS cells (f). (h) Summary of the negative chronotropic changes induced by verapamil. Data are the mean ± SEM. *P < 0.05 and **P < 0.01 compared with 0 mM verapamil.

We next sought to determine whether iPS cell-derived cardiomyocytes are able to respond appropriately to various pharmacological interventions. To this end, beating EBs were plated on multielectrode array (MEA) plates, and extracellular electrograms were recorded from several electrodes beneath the beating EBs following exposure of the beating EBs to the *β*-adrenergic receptor agonist isoproterenol (Figures 6(a)–6(d)) and the L-type calcium channel blocker verapamil (Figures 6(e)–6(h)). Isoproterenol dose-dependently increased the spontaneous beating frequency of iPS cell-derived cardiomyocytes (Figure 6(d)). The beating frequency of Nanog-iPS cell-derived cardiomyocytes was increased from 1.1 ± 0.1 to 1.6 ± 0.2 Hz in the absence and presence of 500 nM isoproterenol, respectively (*n* = 3; *P* < 0.01), whereas that of Fbx15-iPS cell-derived cardiomyocytes was increased from 0.5 ± 0.1 to 1.1 ± 0.5 Hz (*n* = 3; *P* < 0.01). Similar responses were observed to 500 nM isoproterenol in ES cell-derived cardiomyocytes (Figure 6(d)). In contrast, verapamil dose-dependently decreased the frequency of spontaneous beating (Figure 6(h)). The beating frequency of Nanog-iPS cell-derived cardiomyocytes was reduced from 1.5 ± 0.3 to 1.1 ± 0.1 Hz in the absence and presence of 1000 nM verapamil, respectively (*n* = 3; *P* < 0.01), whereas that of Fbx15-iPS cell-derived cardiomyocytes was reduced from 0.6 ± 0.1 to 0.4 ± 0.1 Hz (*n* = 3; *P* < 0.01). Similar responses were observed to 1000 nM verapamil in ES cell-derived cardiomyocytes (Figure 6(h)). These experiments demonstrate that both Nanog-iPS and Fbx15-iPS cell-derived cardiomyocytes have normal chronotropic responses.

## 4. Discussion

A recent breakthrough in pluripotent stem cell technology has opened up the possibility of using iPS cells in patient-specific stem cell therapy. Since the first report from Takahashi and Yamanaka, many contributions have been made to this emerging field [9]. Many delivery systems of reprogramming transcription factors can be used to generate iPS cells [9–12, 25–31]. In addition, various tissues can be used to generate iPS cells, including embryonic fibroblasts, adult dermal fibroblasts, hepatocytes, stomach cells, neural stem cells, adipose stem cells, and hematopoietic stem/progenitor cells [14, 32–36]. The molecular characteristics and the quality of individual iPS cell lines may differ depending on the reprogramming strategy used and the origin of the cells, and it is important to identify these differences before the clinical application of these cells [21, 37–39].

In the current study, we used the Nanog-iPS cells and Fbx15-iPS cells. We observe many distinct characters among those iPS cells (Supplementary Table 2). The timing of initial iPS cell colony generation could also affect the quality of iPS cells in terms of transgene silencing, chromosomal stability, and germline contribution [40]. They were generated by similar protocols but precise timing of iPS cell generation may differ, which may affect the quality of iPS cells. The iPS cell-derived cardiomyocytes displayed typical cardiac phenotypes, including expression of cardiac-specific transcription factors and proteins, and typical electrophysiological properties. Although the structural and functional properties of iPS cell-derived cardiomyocytes were similar, there were obvious differences between the Nanog-iPS and Fbx15-iPS cells in terms of spontaneous beating efficiency and the temporal expression of cardiac-specific transcription factors and proteins. Because of the large numbers of mature cardiac myocytes needed in cardiac regenerative medicine, it is better to use high-quality iPS cells (Nanog-iPS cells in the present study). The Fbx15-iPS cells retained residual transgene expression, and even after purification of Fbx15-iPS cell-derived cardiomyocytes, the expression of residual transgenes remained relatively high. It remains unclear if the residual transgene expression would directly affect the cardiac differentiation efficiency and if the suppression of transgene in low quality iPS cells would increase the cardiac differentiation efficiency. There is another possibility that residual transgene expression is simply a marker for low quality iPS cells, and low quality iPS cells would show low cardiac differentiation efficiency. In addition, the risk of tumor formation persists after transplantation of such cells, and the residual expression of transgenes may hamper normal cardiomyocyte function.

## 5. Conclusions

Prior to the future clinical application of these cells in cardiac regenerative medicine, it is imperative that iPS cells which will be used for regenerative therapy can generate sufficient numbers of cardiomyocytes with minimal risk. The results of the present study raise safety concerns regarding the selection of iPS cell lines and advancing these cells into clinical practice in the future.

## Supplementary Material

Supplementary Table 1. The table shows the primers for the experiment in Fig. 1-4.
Supplementary Table 2. This table summarizes the characteristic differences between ES, Nanog-iPS, and Fbx15-iPS cells as well as cardiomyocytes derived from these cells.
