# Supplementary material for: Distinct iPS Cells Show Different Cardiac Differentiation Efficiency
Source: Stem Cells Int. 2013 Oct 27;2013:659739. doi: 10.1155/2013/659739 (PMC3842496; doi:10.1155/2013/659739)
Supplement: Supplementary file 1 — Supplementary Table 1. The table shows the primers for the experiment in Fig. 1-4. Supplementary Table 2. This table summarizes the characteristic differences between ES, Nanog-iPS, and Fbx15-iPS cells as well as cardiomyocytes derived from these cells. [file 659739.f1.docx]

**Supplementary Table 1**

|  | Sense primers | Antisense primers |
| --- | --- | --- |
| Oct3/4 transgene | TTGGGCTAGAGAAGGATGTGGTTC | TTATCGTCGACCACTGTGCTGCTG |
| Sox2 transgene | GGTTACCTCTTCCTCCCACTCCAG | TTATCGTCGACCACTGTGCTGCTG |
| Klf4 transgene | GCGAACTCACACAGGCGAGAAACC | TTATCGTCGACCACTGTGCTGCTG |
| c-Myc transgene | CAGAGGAGGAACGAGCTGAAGCGC | TTATCGTCGACCACTGTGCTGCTG |
| Oct3/4 endogeneous | TCT TTC CAC CAG GCC CCC GGC TC | CTG TAG GGA GGG CTT CGG GCA CTT |
| Sox2 endogeneous | TAG AGC TAG ACT CCG GGC GAT GA | TTG CCT TAA ACA AGA CCA CGA AA |
| Klf4 endogeneous | GCG AAC TCA CAC AGG CGA GAA ACC | TCG CTT CCT CTT CCT CCG ACA CA |
| c-Myc endogeneous | TGA CCT AAC TCG AGG AGG AGC TGG AAT C | AAG TTT GAG GCA GTT AAA ATT ATG GCT GAA GC |
| Actc1 | CTGAGATGTCTCTCTCTCTCTTAG | ACAATGACTGATGAGAGATG |
| Myh6 | GGAAGAGTGAGCGGCCATCAAGG | CTGCTGGAGAGGTTATTCCTCG |
| Myh7 | GCCAACACCAACCTGTCCAAGTTC | TGCAAAGGCTCCAGGTCTGAGGGC |
| Myl7 | CAGACCTGAAGGAGACCT | GTCAGCGTAAACAGTTGC |
| Myl2 | GCCAAGAAGCGGATAGAAGG | CTGTGG TTCAGGGCTCAGTC |
| Nppb | ATGGATCTCCTGAAGGTGCT | TCTTGTGCCCAAAGCAGCTT |
| GAPDH | TTCAACGGCACAGTCAAGG | CATGGACTGTGGTCATGA G |

**Supplementary Table 2**

TaqMan probe for Quantitative RT-PCR

| Brachyury T | Mm00436877_m1 |
| --- | --- |
| Mesp1 | Mm00801883_g1 |
| Nkx2.5 | Mm00657783_m1 |
| Gata4 | Mm00484689_m1 |
| Nppa | Mm011255747_g1 |
| Myl2 | Mm00440384_m1 |
